# Comparison of the Fungistatic Activity of Selected Essential Oils Relative to *Fusarium graminearum* Isolates

**DOI:** 10.3390/molecules24020311

**Published:** 2019-01-16

**Authors:** Teresa Krzyśko-Łupicka, Weronika Walkowiak, Marietta Białoń

**Affiliations:** 1 Department Biotechnology and Molecular Biology, University of Opole, ul. Kard. B. Kominka 6a, 45-032 Opole, Poland; weronika.walkowiak@gmail.com; 2 Faculty of Chemistry, University of Opole, Oleska 48, 45-052 Opole, Poland

**Keywords:** *Fusarium graminearum*, essential oils, geranium oil, rosewood oil, lemon oil, rosemary oil

## Abstract

The aim of the study was to determine the chemical composition of lemon, rosewood, geranium and rosemary oils, and compare their effect on the sensitivity of *Fusarium graminearum* ZALF 24 and *Fusarium graminearum* ZALF 339 isolated from infected cereals. The tested oils were added to Potato Dextrose Agar (PDA) medium at concentrations of 0.125%, 0.25%, 0.5%, 1.0% and 2.0%. The activity of the oils on inhibition of the linear growth of mycelium was evaluated by measuring the growth of fungal colonies (growth index), while the fungistatic activity was evaluated on the basis of the percentage growth inhibition of a fungal colony and calculated according to Abbott’s formula. The sensitivity of the test strains was variable and depended on the type and concentration of the tested oils. Geranium and rosewood oils in all of the concentrations completely inhibited the growth of the used isolates. In contrast, lemon oil relative to *F. graminearum* ZALF 339 showed the highest activity at a concentration of 1.0% and rosemary oil, 0.5%. The highest activity against *F. graminearum* ZALF 24 was shown by the oils of rosemary and lemon at concentrations from 1.0% to 2.0%. The susceptibility of *Fusarium graminearum* isolates was differentiated and depended on the type and concentration of tested oils.

## 1. Introduction

In Poland, since January 2014, there has been an obligation to apply the principles of integrated cropping systems in agriculture, resulting from the provisions of Art. 14 of Directive 2009/128/EC and Regulation No. 1107/2009. These principles involve a reduction in the consumption of chemical pesticides and fertilisers. Instead, appropriate agricultural practices, organic fertilisation, and biological methods, which use microbial activity as well as biologically active substances, should be applied.

Both in the case of an integrated system of cultivation and organic waste management, there are few technologies available that can improve productivity and increase competitiveness on the market. Chemical fungicides, such as Cuprate 50 HR, Amistar 250 SC, Tango Star 334 SE, Caramba 60 SL and Folicur Plus 375 EC Artea 330 EC [1], which are currently used in the cultivation of plants to combat phytopathogenic fungi of the genus *Fusarium*, despite their efficiency and simplicity of application, cause a risk to the health and safety of the consumer. The threat is caused by fungicide residues, increasing immunisation of fungal pathogens, and the reduction of beneficial organisms [2,3]. Synthetic fungicides, which are nonbiodegradable in the environment, have a negative impact on terrestrial ecosystems, which is why a biological control seems to be a good alternative to chemicals [4,5].

The alternative could be essential oils that show fungicidal activity [6] and are safe for people and the environment [7]. Numerous studies have shown that essential oils that are a mixture of monoterpenes, monoterpenoids, sesquiterpenes and fragrances (esters, ketones, phenols, alcohols, aldehydes, ethers, hydrocarbons, coumarins and organic acids) effectively limit the development of phytopathogenic fungi types: *Fusarium* (*Fusarium oxysporum*, *Fusarium culmorum*), *Phytophthora*, *Stemphylium*, *Sphaerotheca*, *Botrytis*, *Erysiphe*, *Aspergillus*, *Mortierella*, *Sclerotinia*, *Sporotrichum*, *Penicillium* and *Alternaria* [8,9,10] and yeast [11]. The effect of their biocidal action is dependent on their chemical composition and the essential oils’ concentration, as well as the sensitivity of phytopathogenic fungi strains; thus, research into the use of essential oils as effective biofungicides is constantly conducted.

The aim of the study was to determine the chemical composition of lemon, rosewood, geranium and rosemary oils and to compare their effect on the sensitivity of *Fusarium graminearum* ZALF 24 and *Fusarium graminearum* ZALF 339 isolated from infected cereals.

## 2. Results

**Figure A1:**
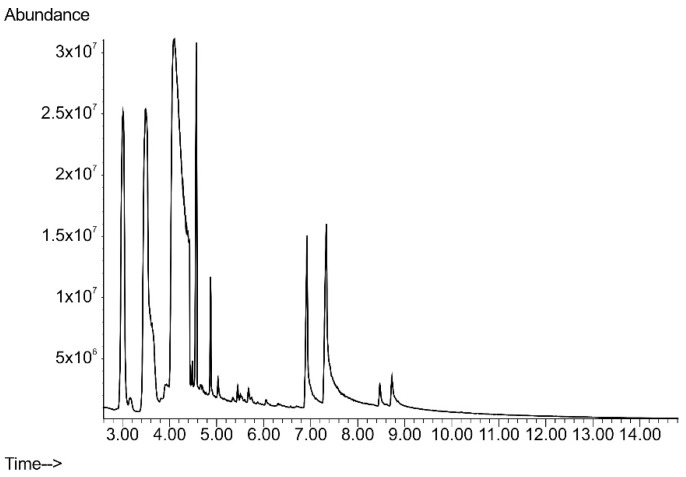
The gas chromatogram of the lemon commercial oil. Dilution 1:40 (*v*/*v*) in dichloromethane.

**Figure A2:**
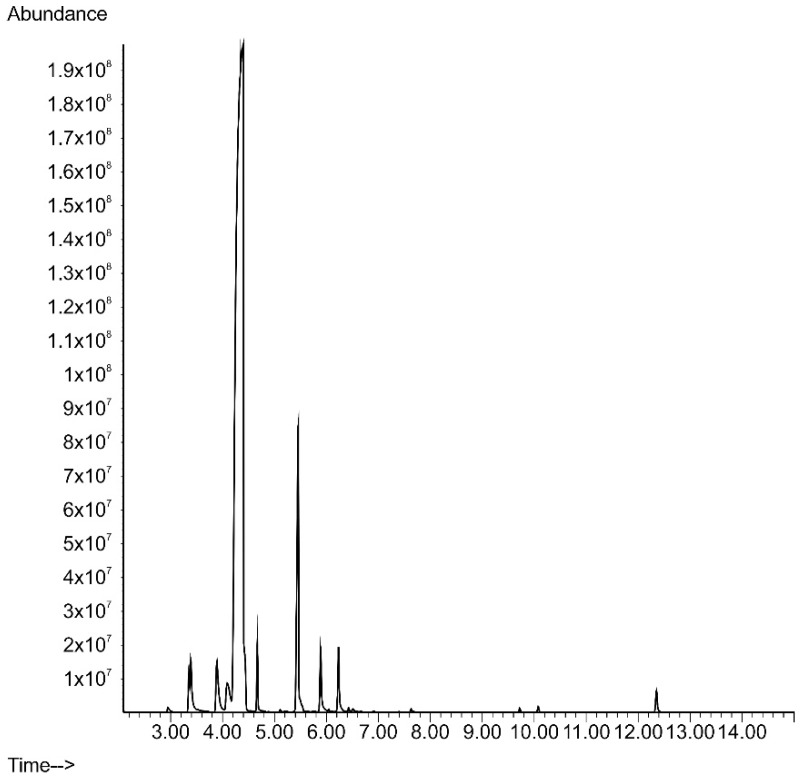
The gas chromatogram of the rosewood commercial oil. Dilution 1:40 (*v*/*v*) in dichloromethane.

**Figure A3:**
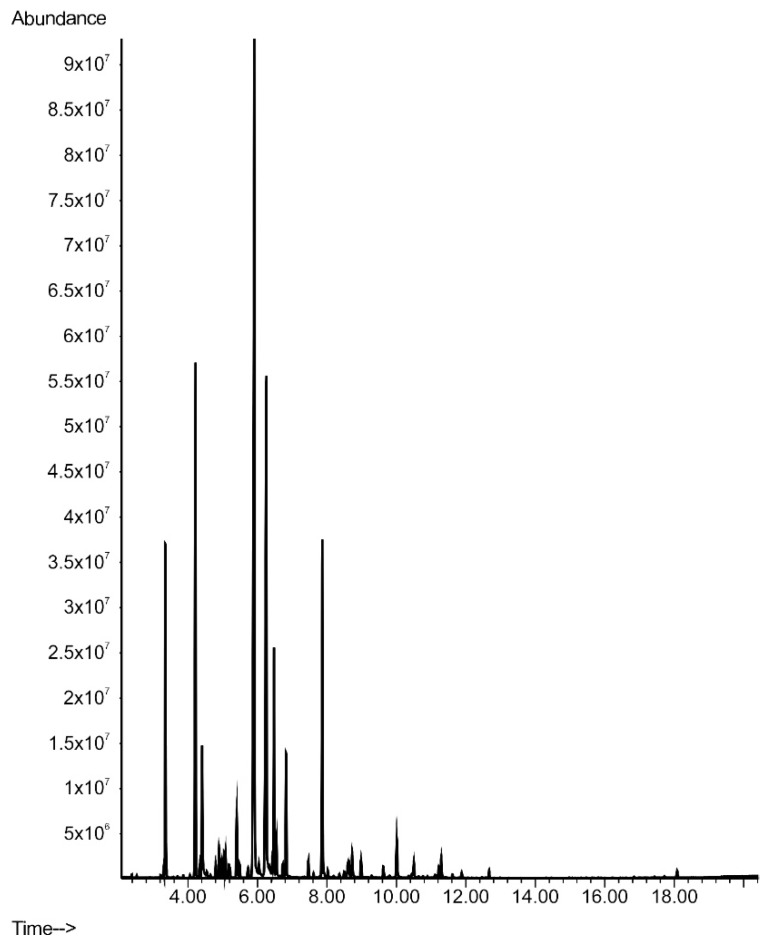
The gas chromatogram of the geranium commercial oil. Dilution 1:40 (*v*/*v*) in dichloromethane.

**Figure A4:**
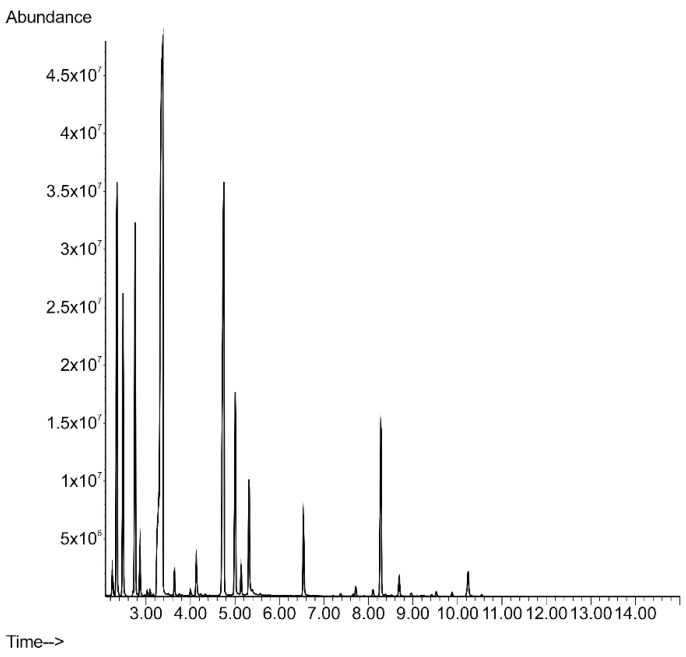
The gas chromatogram of the rosemary commercial oil. Dilution 1:40 (*v*/*v*) in dichloromethane.

In studies on the reduction of the development of phytopathogens, essential oils of different chemical composition were used. Table 1 and Table 2 present the main types of terpenes found in the tested oils and the details of the chemical composition. The chromatograms of the used essential oils are presented in Figure A1, Figure A2, Figure A3 and Figure A4. The rosemary and geranium oils contained mainly monoterpenoid (over 96% and over 88%, respectively), whereas lemon oil contained mainly monoterpenes (less than 86%) and only 13.8% monoterpenoids. The most varied in terms of chemical composition turned out to be rosemary oil, which contained both monoterpenoids (52.8%) and monoterpenes (35.3%) as well as sesquiterpenes (11.5%).

The geranium and rosemary essential oils contained 56 and 33 terpenes and terpenoides, respectively. In contrast, the lemon and rosewood oils contained only 20 terpene compounds. On the basis of the GCMS analysis, it was found that *β*-citronellol, geraniol and linalool were present in geranium essential oils in the largest amounts, respectively 31%, 17.2% and 11.3%. The following compounds were the main components of the rosemary essential oil: *α*-phellandren-8-ol (16%), eucalyptol (15.9%) and *α*-pinene (10.3%). In the lemon essential oil, the main compounds were limonene (48,3%), *β*-pinene (15.1%) and *α*-pinene (11.1%). Linalool (79.5%) and *α*-terpineol (8,3%) were the main components of the rosewood essential oil (Table 2).

All tested oils, when compared to a relative control at concentrations of 0.125% to 2.0%, decreased the mycelial growth of *F. graminearum*. The growth index of *F. graminearum* ZALF 24 and *F. graminearum* ZALF 339 was differentiated and it depended on the type and concentration of the oil used (Table 3 and Table 4).

**Figure A5:**
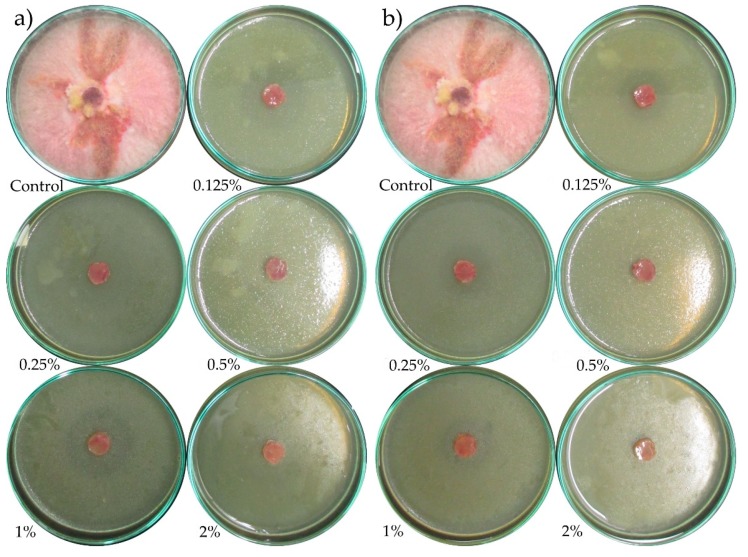
The effect of geranium oil concentrations on the growth of *Fusarium graminearum* ZALF 24 (**a**) and *Fusarium graminearum* ZALF 339 (**b**).

**Figure A6:**
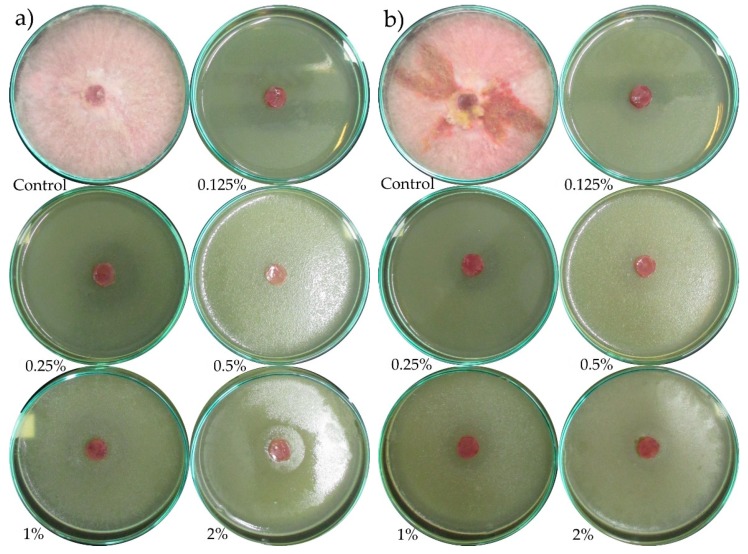
The effect of rosewood oil concentrations on the growth of *Fusarium graminearum* ZALF 24 (**a**) and *Fusarium graminearum* ZALF 339 (**b**).

Complete inhibition of mycelium growth, regardless of the concentration used, was caused by the geranium and rosewood oils, the active substances of which were oxygenated monoterpenes: citronellol and geraniol in the geranium oil and linalool in the rosewood oil (Table 2 and Table 3, Figure A5a and Figure A6a).

A similar relationship for the geranium and rosewood oils was observed for the strain of *F. graminearum* ZALF 339 (Table 4, Figure A5b and Figure A6b).

After 11 days of incubation, the geranium oil completely inhibited the growth of the tested strains at all examined concentrations; the same relationship can be observed for the essential oil of rosewood.

**Figure A7:**
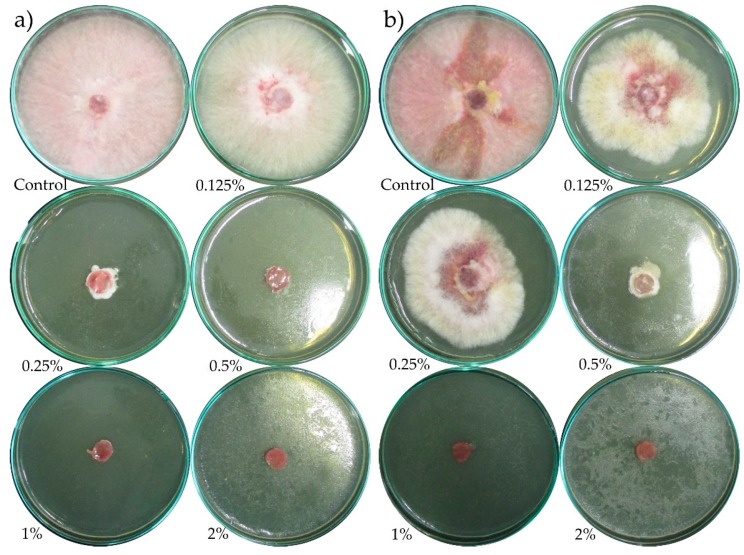
The effect of lemon oil concentrations on the growth of *Fusarium graminearum* ZALF 24 (**a**) and *Fusarium graminearum* ZALF 339 (**b**).

**Figure A8:**
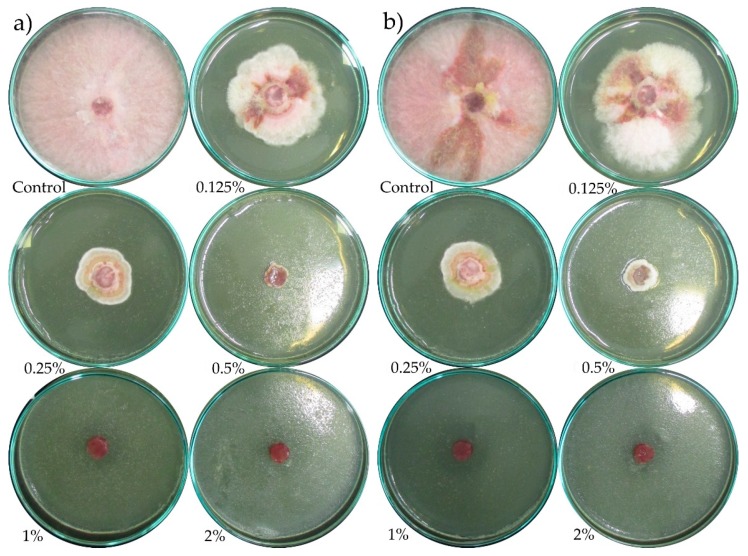
The effect of rosemary oil concentrations on the growth of *Fusarium graminearum* ZALF 24 (**a**) and *Fusarium graminearum* ZALF 339 (**b**).

The rosemary and lemon oils, despite the differences in the chemical composition, worked similarly on the tested *Fusarium* isolates. The total inhibition of linear mycelium growth was observed in both cases only after applying oils at 1 and 2% concentrations, for example (Table 3 and Table 4, Figure A7 and Figure A8).

In contrast, the index of linear growth of the test strains in the presence of lemon oil varied and depended on the concentration used. The oil at a concentration of 0.125% showed little effect on the inhibition of linear growth of the tested fungi. The increase of lemon oil concentration, depending on the strains, inhibited the growth of these fungi in different ways. The development of the mycelium *F. graminearum* ZALF 24 was completely inhibited by the oil at concentrations of 0.5% to 2.0% (Table 3) and *F. graminearum* ZALF 339 at a concentration of 1.0% to 2.0% (Table 4).

Fungistatic activity also depended on the oil concentration and strain sensitivity. The highest fungicidal activity with respect to both tested strains of *F. graminearum*, regardless of the concentration used, was shown by the geranium and rosewood oil. The total inhibition of the growth of tested fungi was observed in the entire concentration of both of these essential oils.

**Figure 1:**
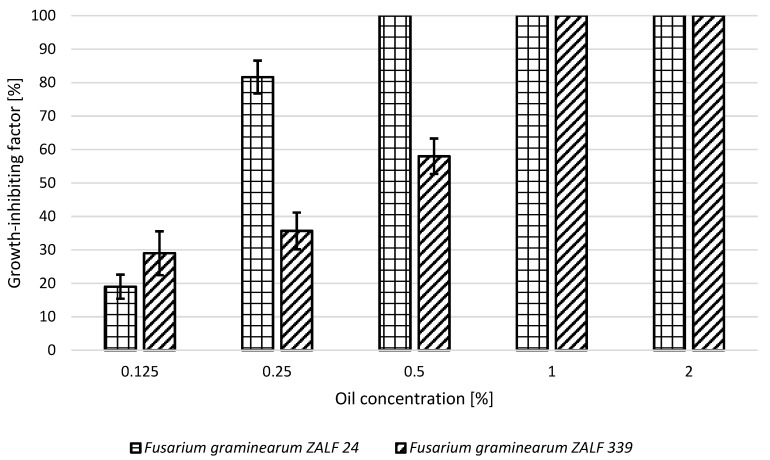
Growth-inhibiting factor of *Fusarium graminearum* ZALF 24 and *Fusarium graminearum* ZALF 339 strains in the presence of lemon oil.

In contrast, the highest fungicidal activity of lemon oil was observed in *F. graminearum* ZALF 24 at a concentration of 0.5% to 2.0%, and in *F. graminearum* ZALF 339 at a concentration of 1.0% to 2.0%. At lower concentrations, the fungistatic activity of lemon oil ranged from 19% to 82% for *F. graminearum* ZALF 24 and from 29 to 58% for *F. graminearum* ZALF 339 (Figure 1).

**Figure 2:**
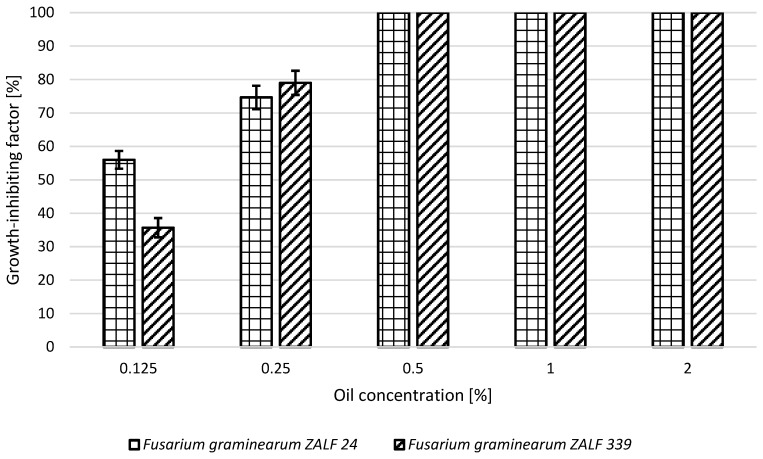
Growth-inhibiting factor of *Fusarium graminearum* ZALF 24 and *Fusarium graminearum* ZALF 339 strains in the presence of rosemary oil.

At lower concentrations, the fungistatic activity of the rosemary oil ranged from 56% to 75% for *F. graminearum* ZALF 24 and from 36% to 78% for *F. graminearum* ZALF 339. It showed the highest fungistatic activity at concentrations from 0.5% to 2.0% (Figure 2).

## 3. Discussion

Phytopathogenic species of *Fusarium graminearum* are found in nature in the heterothallic and homothallic forms. Distinctive heterothallic strains create a fluffy and airy mycelium with colouring from white to yellow or light brown on Potato Dextrose Agar (PDA) medium and occur mostly in Australia and California. In contrast, homothallic strains, which are located in Europe, including Poland, and the eastern part of the United States, create a sparser airy mycelium of pink carmine colour with a shade of yellow [12]. The sensitivity of fungi strains of the *Fusarium* genus to essential oils may already vary within the species and may depend on both the strain and the chemical composition, as well as on the concentration of essential oils. The qualitative and quantitative composition of the active substance contained in the raw material decides the biological activity and efficacy of essential oils [13,14,15,16], but even a small percentage of another compound in the oil may affect the fungicidal activity [17]. Differentiation of the various components contained in the essential oils has an impact on their properties and bioactivity [18].

*F. graminearum* sensitivity to essential oils is the subject of many studies [19,20,21,22,23].

In our studies, we found that all tested essential oils had fungicidal activity, including the total inhibition of mycelial growth of *F. graminearum* ZALF 24 and *F. graminearum* ZALF 339, regardless of the concentration of the used geranium and rosewood oil. A high fungistatic activity of these oils was also exhibited by other researchers [24,25,26]. These oils worked similarly despite clear differences in their chemical composition. The active substances of geranium oil are citronellol and geraniol, while linalool is active in rosewood oil. For this reason, they can be the basis for constructing biofungicides.

Other tested oils showed the desired effect at higher concentrations. The highest biocidal activity of the rosemary oil on the studied strains (*F. graminearum* ZALF 24 and *F. graminearum* ZALF 339) was observed for a concentration higher than 0.5%. The fungicidal properties of rosemary oil with respect to *Fusarium* are confirmed by the studies of Dimitra et al. [18] and Surviliené et al. [10]. Dimitra et al. [18] suggested that borneol, not eucalyptol (the major component of this oil), is likely to be responsible for its fungistatic activity. However, Ćosić et al. [27] have shown that rosemary oil, as well as cinnamon, sage, pine, bitter orange, anise, cumin and lavender oils, did not inhibit the growth of an *F. graminearum* strain. Only a strong biocidal effect in relation to this fungus was observed in thyme oil, and a weak one in peppermint oil. It is likely that the obtained results depended on the methods used, the concentrations of essential oils and the form of the occurring strains (heterothallic and homothallic).

The studies also demonstrated the efficacy of lemon oil at concentrations of 0.5–2.0% in reducing the development of *F. graminearum* strains. However, this was not confirmed in the research of Gömöri et al. [28], although Viuda-Martos et al. [17] showed that this oil at the concentration of 0.94% completely inhibited the growth of *Aspergillus niger*, *Aspergillus flavus*, *Penicillium verrucosum* and *Penicillium chrysogenum* fungi. Antifungal properties of citrus oils are attributed to the presence of such components as D-limonene and linalool; however, even a small percentage of another compound in the oil may affect its fungicidal activity [29]. The main ingredient of the tested lemon oil was D-limonene; however, smaller quantities of citral, *α*-terpineol, *α*-pinene, *β*-pinene, citronellal, linalyl and geranyl acetate, p-cymene, *γ*-pinene, *β*-myrcene, coumarins and bioflavonoids occurring in this oil could have had an effect on the biocidal properties. A proper selection of essential oils exhibiting fungicidal activity even at low concentrations can be used in biological plant protection against phytopathogens of the *Fusarium* genus, and seem to be a good alternative to chemicals [4,5,6,7].

## 4. Materials and Methods

### 4.1. Materials

The research material was strains of *Fusarium graminearum* isolated on PDA medium from infected cereals. The strains were identified on the basis of morphological characteristics [12,30,31]. Commercial essential oils (produced by ETJA, Elbląg, Poland), such as geranium (*Pelargonium graveolens*), rosewood (*Aniba Rosaeodora*), rosemary (*Rosmarinus officinalis*) and lemon (*Citrus limonum*), which are widely available in the course of trade, were tested.

### 4.2. The Gas Chromatography Mass Spectrometry Analysis

The Hewlett Packard HP 6890 series GC system chromatograph (Hewlett Packard, WALDBRONN, Germany) was used for the study, which was coupled with the Hewlett Packard 5973 mass selective detector (Hewlett Packard, Waldbronn, Germany). The chromatograph was equipped with the non-polar, high-temperature ZB-5HT capillary column; length, 30 m; inner diameter, 0.32 mm; film thickness, 0.25 μm (Phenomenex Inc., Torrance, CA, USA). The on-column injector was used and 1 μm of a sample was introduced. The initial temperatures, both of the injector and the oven, were 60 °C, and the temperature was increased by 10 °C per minute up to 280 °C; the auxiliary temperature was 300 °C. Helium was used as the carrier gas and its flow was 2 mL/min. The components were identified by comparison of their mass spectra with the spectrometer database of the NIST 11 Library (National Institute of Standards and Technology, Gaithersburg, MD, USA) and by comparison of their retention index calculated against n-alkanes (C_9_–C_20_). Each chromatographic analysis was repeated three times. The average value of the relative composition of the essential oil percentage was calculated from the peak areas.

### 4.3. Biological

Tested oils were inserted into a PDA medium (Potato Dextrose Agar produced by BioMaxima S.A., Lublin, Poland) enriched with 0.01% Tween 80 (produced by BTL, Warsaw, Poland) in the following concentrations: 0.125; 0.25; 0.5; 1.0; 2.0%. The control was carried out through the growth of tested isolates in modified PDA medium (without oils).

The biotic activity of oils in reducing the linear growth of the fungus *F. graminearum* ZALF 24 and *F. graminearum* ZALF 339 was assessed by using the method of poisoned substrates [32]: cultures were grown in PDA medium for 14 days at 25 °C, and inoculum—the spore suspension of *F. graminearum* ZALF 24 and *F. graminearum* ZALF 339 in a 0.01% sterile solution of Tween 80 (produced by BTL, Warsaw, Poland)—was obtained from an 11-day-old culture. The haemocytometer Thoma was used to obtain a spore suspension of 1·10^6^ CFU·cm^3^. Petri dishes (9 cm diameter) containing 20 cm^3^ PDA medium were used to inoculate this spore suspension and stored at 25 °C for 11 days. Inoculum rings with a diameter of 10 mm overgrown by mycelium were obtained. The absolute control was the culture of the fungus on modified PDA medium without oils.

On the basis of measurements of the fungal colony, the linear growth of mycelium index (T) and the fungistatic activity of essential oils were calculated.

The growth rates index of *Fusarium* strains was calculated using the following formula (1) [33]:

(1) $$T=\frac{A}{D}+\frac{b_{1}}{d_{1}}+\ldots +\frac{b_{x}}{d_{x}}$$

where:

*T* – index of linear growth
*A* – average measurement value of diameter colonies (mm)
*D* – duration of the experiment
*b_1_….b_x_* – increase in colonies diameter (mm)
*d_1_..d_x_* – number of days since last measurement

The fungistatic activity of the tested oils was assessed based on the percentage of the growth inhibition of fungus colonies and calculated using Abbott’s formula (2):

(2) $$I=\frac{C-M}{C}100$$

where:

*I* – fungus linear growth inhibition index (%)
*C* – fungus colony diameter in the control combination (mm)
*M* – fungus colony diameter on a control plate with a given oil in the combination containing a tested substance concentration in the medium [mm]

All analyses were performed with three repetitions. A two-way analysis of variance (ANOVA) was used to examine the effect of essential oils. A post-hoc test by Tukey’s HSD was used to determine significant differences at a level of P < 0.05. All statistical analyses were done using R.

## 5. Conclusions

The growth index of *F. graminearum* ZALF 24 and *F. graminearum* ZALF 339 varied and depended on the used oil and its concentration. The highest fungicidal activity with respect to both *F. graminearum* strains, regardless of the concentration, was shown by the geranium oil and the rosewood oil, which contain about 88–97% monoterpenoids. The development of the *F. graminearum* ZALF 24 strain was inhibited by the rosemary and lemon oils at concentrations from 0.5% to 2.0%, and *F. graminearum* ZALF 339 by a 1.0% concentration of lemon oil. All tested oils, compared to the control, reduced the rate of mycelial growth of *Fusarium graminearum* and showed fungistatic activity.

## Figures and Tables

**Table 1:** The contents of terpenes (%) in the tested essential oils.

	Area (%) of Terpenes in Etja Essential Oils			
	Lemon	Rosewood	Geranium	Rosemary
monoterpenes	85.70	0.75	8.09	35.27
oxygenated monoterpenes	13.76	96.75	88.17	52.76
sesquiterpenes	-	-	1.17	11.54
oxygenated sesquiterpenes	-	0.95	1.34	0.30

The remainder up to 100% were non-terpenes compounds.

**Table 2:** The terpene ingredients (%) of the tested essential oils.

	IR	Area (%) ± SD of Components in Etja Essential Oils			
		Lemon	Rosewood	Geranium	Rosemary
MONOTERPENES					
tricyclene	919	0.21 ± 0.04	-	-	0.48 ± 0.03
*α*-pinene	930	11.06 ± 0.14	-	0.05 ± 0.01	10.33 ± 0.03
camphene	946	0.35 ± 0.04	-	0.04 ± 0.01	8.18 ± 0.03
*β*-citronellene	948	-	0.02 ± 0.01	-	-
*β*-thujene	970	-	-	-	3.92 ± 0.20
*β*-pinene	973	15.14 ± 0.04	-	0.03 ± 0.01	7.62 ± 0.14
2,6-dimethyl-2,6-octadiene	980	-	-	0.45 ± 0.01	-
3-menthene	981	-	-	0.26 ± 0.02	-
*β*-myrcene	984	4.11 ± 0.06	-	-	1.72 ± 0.03
2-carene	996	-	-	0.05 ± 0.01	-
*α*-phellandrene	999	-	-	-	0.11 ± 0.02
*α*-terpinene	1016	0.40 ± 0.12	-	-	0.06 ± 0.01
p-cymene	1019	-	-	0.19 ± 0.01	-
limonene	1024	48.27 ± 0.05	0.73 ± 0.02	6.86 ± 0.05	-
trans-*β*-ocimene	1040	0.13 ± 0.03	-	-	-
*α*-ocimene	1050	-	-	0.01 ± 0.01	-
*β*-terpinene	1053	-	-	0.01 ± 0.01	-
*γ*-terpinene	1059	4.85 ± 0.06	-	-	2.16 ± 0.02
terpinolene	1077	1.19 ± 0.03	-	-	0.68 ± 0.02
**Sum**	**-**	**85.70**	**0.75**	**8.09**	**35.27**
**OXYGENATED MONOTERPENES**					
eucalyptol	1030	-	1.55 ± 0.04	-	15.87 ± 0.12
dehydrolinalool	1072	-	0.11 ± 0.01	-	-
cis-linalool oxide	1073	-	2.07 ± 0.03	0.05 ± 0.01	-
rose oil	1087	-	**-**	3.05 ± 0.04	-
trans-linalool oxide	1089	-	1.56 ± 0.03	0.07 ± 0.01	-
cis rose oxide	1096	-	**-**	0.20 ± 0.01	-
linalool	1099	0.29 ± 0.06	79.51 ± 0.32	11.27 ± 0.06	1.83 ± 0.06
myrcenol	1099	-	0.06 ± 0.01	-	-
trans rose oxide	1113	-	**-**	0.09 ± 0.01	-
1-terpineol	1121	-	**-**	0.05 ± 0.01	-
*α*-pinene oxide	1126	0.17 ± 0.02	-	-	-
menthone	1133	-	**-**	0.59 ± 0.02	-
camphor	1141	-	-	-	16.00 ± 0.11
*α*-phellandren-8-ol	1144	-	0.01 ± 0.01	-	-
isomenthone	1144	-	**-**	0.53 ± 0.03	-
isoborneol	1145	-	**-**	0.32 ± 0.01	-
isopulegol	1149	-	**-**	0.33 ± 0.01	-
verbenol	1154	0.08 ± 0.01	-	-	-
menthol	1164	0.12 ± 0.01	-	-	-
borneol	1168	-	-	0.49 ± 0.01	7.95 ± 0.10
lavandulol	1171	-	1.72 ± 0.01	-	-
terpinen-4-ol	1174	-	-	-	1.43 ± 0.03
*γ*-terpineol	1185	-	**-**	0.32 ± 0.03	-
*α*-terpineol	1197	-	8.27 ± 0.01	1.53 ± 0.03	5.62 ± 0.12
fenchol	1199	-	-	-	0.08 ± 0.01
*α*-fenchyl acetate	1202	-	-	-	0.50 ± 0.05
linalyl formate	1205	-	-	2.81 ± 0.09	-
*β*-citronellol	1208	-	-	30.99 ± 0.20	-
*α*-citronellol	1209	0.20 ± 0.02	-	4.73 ± 0.23	-
nerol	1232	-	1.64 ± 0.02	-	-
geraniol	1233	-	-	17.20 ± 0.10	-
*α*-citral	1247	7.14 ± 0.07	0.08 ± 0.01	0.32 ± 0.03	-
2,6-dimethyl-1,7-octadiene-3,6-diol	1268	-	0.07 ± 0.01	-	-
borneol acetate	1270	-	-	-	3.47 ± 0.03
*β*-citral	1278	4.30 ± 0.14	0.05 ± 0.01	0.25 ± 0.11	-
geranyl formate	1294	0.91 ± 0.01	-	0.62 ± 0.03	-
citronellol acetate	1338	-	-	0.35 ± 0.01	-
p-mentha-1-en-3,8-diol	1351	-	0.03 ± 0.01	-	-
neryl acetate	1364	0.49 ± 0.06	-	0.51 ± 0.01	-
geranyl acetate	1383	-	-	8.56 ± 0.22	-
cis-geranylacetone	1418	-	-	0.17 ± 0.01	-
carvone hydrate	1427	-	0.02 ± 0.01	-	-
citronellyl propionate	1431	-	-	0.33 ± 0.02	-
geranyl propionate	1451	-	-	0.10 ± 0.01	-
geranyl isobutyrate	1494	-	-	0.70 ± 0.01	-
geranyl butyrate	1542	-	-	1.22 ± 0.03	-
citronellyl tiglate	1646	-	-	0.30 ± 0.03	-
geranyl tiglate	1657	-	-	0.12 ± 0.01	-
**sum**	**-**	**13.76**	**96.75**	**88.17**	**52.76**
**SESQUITERPENES**					
*α*-cubebene	1354	-	**-**	-	0.16 ± 0.01
*α*-longipinene	1359	-	**-**	-	0.07 ± 0.01
*α*-copaene	1379	-	**-**	0.03 ± 0.01	0.57 ± 0.02
*β*-cubebene	1384	-	**-**	-	0.20 ± 0.05
*β*-bourbonene	1388	-	**-**	0.10 ± 0.02	-
y-langene	1388	-	**-**	-	0.15 ± 0.02
*γ*-maaliene	1398	-	**-**	0.03 ± 0.01	-
aristolene	1402	-	**-**	0.38 ± 0.02	-
longifolene	1421	-	**-**	0.05 ± 0.00	0.57 ± 0.01
caryophyllene	1423	-	**-**	0.11 ± 0.01	7.58 ± 0.03
alloaromadendrene	1426	-	**-**	0.05 ± 0.01	-
calarene	1436	-	**-**	0.16 ± 0.01	-
aromadendrene	1443	-	**-**	-	0.08 ± 0.01
humulene	1452	-	**-**	-	1.36 ± 0.08
ledene	1470	-	**-**	0.22 ± 0.01	-
*γ*-muurolene	1479	-	**-**	-	0.11 ± 0.01
isocaryophyllene	1495	-	-	-	0.04 ± 0.01
*γ*-cadinene	1503	-	**-**	-	0.29 ± 0.03
*δ*-selinene	1510	-	**-**	0.04 ± 0.01	-
*δ*-cadinene	1525	-	**-**	-	0.37 ± 0.02
**Sum**	**-**	**-**	**-**	**1.17**	**11.54**
**OXYGENATED SESQUITERPENES**					
elemol	1535	-	-	0.03 ± 0.01	-
nerolidol	1540	-	0.11 ± 0.01	-	-
cis-nerolidol	1545	-	0.16 ± 0.02	-	-
trans-nerolidol	1551	-	0.68 ± 0.02	-	-
guaiol	1584	-	-	0.47 ± 0.02	-
caryophyllene oxide	1588	-	-	-	0.30 ± 0.02
*γ*-eudesmol	1620	-	-	0.04 ± 0.01	-
bulnesol	1650	-	-	0.54 ± 0.03	-
methyl abietate	2175	-	-	0.26 ± 0.03	-
**Sum**		**-**	**0.95**	**1.34**	**0.30**

IR, Kovats retention index; SD, standard deviation. The remainder up to 100% were non-terpenes compounds.

**Table 3:** The index of linear growth (T) of *Fusarium graminearum* ZALF 24.

Oil Concentration (%)		Etja Essential Oils			
	Control	Lemon	Rosewood	Geranium	Rosemary
0.125	85.00 a	69.00 b	0.00 f	0.00 f	36.67 c
0.25	85.00 a	15.00 de	0.00 f	0.00 f	20.67 d
0.5	85.00 a	0.00 f	0.00 f	0.00 f	7.33 ef
1.0	85.00 a	0.00 f	0.00 f	0.00 f	0.00 f
2.0	85.00 a	0.00 f	0.00 f	0.00 f	0.00 f

a–f—values denoted with the same letters do not differ statistically (*p* < 0.05).

**Table 4:** The index of linear growth (T) of *Fusarium graminearum* ZALF 339.

Oil Concentration (%)		Etja Essential Oils			
	Control	Lemon	Rosewood	Geranium	Rosemary
0.125	85.00 a	59.67 b	0.00 f	0.00 f	54.00 bc
0.25	85.00 a	54.33 b	0.00 f	0.00 f	19.00 de
0.5	85.00 a	35.67 cd	0.00 f	0.00 f	4.17 ef
1.0	85.00 a	0.00 f	0.00 f	0.00 f	0.00 f
2.0	85.00 a	0.00 f	0.00 f	0.00 f	0.00 f

a–f—values denoted with the same letters do not differ statistically (*p* < 0.05).
